# Super-Selective Transarterial Chemoembolization with Doxorubicin-Loaded Drug-Eluting Beads Sized Below and Above 100 Microns in Hepatocellular Carcinoma: A Comparative Study

**DOI:** 10.5334/jbsr.1841

**Published:** 2019-07-29

**Authors:** Huseyin Balli, Erol Aksungur, Behruz Khalatai, Kairgeldy Aikimbaev

**Affiliations:** 1 Cukurova University, TR

**Keywords:** Chemoembolization, Doxorubicin, Microspheres, Drug-eluting beads, Hepatocellular carcinoma

## Abstract

Objectives To compare efficacy and safety of super-selective DEB-TACE with doxorubicin-loaded microspheres sized below and above 100 microns for treatment of hepatocellular carcinoma (HCC). Material and methods All consecutive patients with HCC who underwent DEB-TACE were included in this retrospective study. Regarding to microsphere size (>100 microns or <100 microns), patients were determined as Group A (n = 28) and Group B (n = 30), respectively. Results Of the 58 patients (78% males), no statistically significant difference was found between the two groups in terms of age and gender (P = 0.388, P = 0.888, respectively). There were no significant differences between the two groups in terms of BCLC stages, presence of chronic liver disease, and Child-Pugh classes (P = 0.593, P = 0.081, P = 0.391, respectively). Although statistically insignificant, median overall survival (19 months vs 32 months, P = 0.190) and median progression-free survival (13 months vs 20 months (P = 0.574) were longer and 1-3-years objective response rates (7.40% vs 23.33%, P = 0.330) were higher in Group B than in Group A, respectively. No mortality or major complications were observed. Grade I/ II adverse events were detected in all patients. Transient elevations in liver function tests (Grade III adverse events) were similar in both groups (3.57% vs 3.33%; P = 0.980). Conclusion Super-selective DEB-TACE with doxorubicin-loaded microspheres sized <100 microns is an effective and safe method for the HCC treatment. Objective response rates are higher and survival durations are longer after DEB-TACE performed with doxorubicin-loaded microspheres sized below 100 microns. Keywords Chemoembolization Doxorubicin Microspheres Drug-eluting beads Hepatocellular carcinoma

## Introduction

Hepatocellular carcinoma (HCC) is the most common primary malignant tumor of the liver, the fifth most common malignant tumor in the world and the third most common cause of cancer-related death worldwide [1]. Only 15–20% of the patients can be diagnosed at early stages, and for these patients, curative treatments such as liver resection, transplantation and local ablation may be implemented. However, the majority of the patients are diagnosed in the intermediate or advanced stages, and therefore only palliative treatments such as transarterial chemoembolization (TACE), transarterial radioembolization or systemic therapy apply [2,3].

In TACE, tumor-feeding arteries are selectively catheterized and carrier microspheres with chemotherapeutic drugs are given. In randomized trials, TACE has been shown to prolong the median survival by 16 to 20 months compared to the best supportive treatments [4,5]. TACE technique is divided into two types: conventional chemoembolization with lipophilic embolic agents (lipiodol) and chemoembolization with drug-eluting beads (DEB) [4]. Studies have shown that DEB-TACE can deliver chemotherapeutic drugs to smaller arteries and increase the concentration of the drug in the tumor compared to conventional TACE. As a result, the necrotizing effect on the tumor is higher with decreasing the side effects to other organs [6]. However, no consensus has been established regarding the optimal size of the microspheres used [7]. Recently, ability for particle selection has increased with the introduction of smaller microspheres such as 30–60 microns, 40–100 microns and 70–150 microns [8,9]. In animal experiments, it has been shown that small microspheres convey the drug more concomitantly and better obliterate the tumor [10]. However, there are few published human studies on the comparison of DEB-TACE using microspheres smaller than 100 microns and using microspheres larger than 100 microns in the treatment of HCC [11,12].

The aim of this study was to compare the overall survival, response of the target lesion to the treatment and minor/major complications in HCC patients ineligible for surgical treatment who underwent DEB-TACE using doxorubicin-loaded microspheres sized below and above 100 microns.

## Materials and methods

This single-center study was conducted retrospectively in accordance with the Declaration in Helsinki by obtaining the approval of the Medical Faculty Research and Ethics Committee (decision from March 2, 2018; approval number 68). Fifty-eight HCC patients who were not eligible for curative treatment and underwent DEB-TACE procedure after the consensus of interdisciplinary tumor board between November 2007 and November 2015 were included in the study. The TACE procedure was performed on patients meeting the following inclusion criteria: Child-Pugh class A or B, patent portal/hepatic veins, HCC involving less than 50% of the liver, Eastern Cooperation Oncology Group (ECOG) performance status ≤ 2, serum creatinine < 2 mg/dL, platelet count > 5000/mm^3^, leukocyte count > 3000/mm^3^, left ventricular ejection fraction > 50%. Exclusion criteria for DEB-TACE procedure were: Child-Pugh class C, ECOG performance status > 2, extrahepatic metastasis, portal or hepatic vein thrombosis, HCC involving more than 50% of the liver, biliary obstruction, chronic renal failure, and congestive heart failure.

DEB-TACE procedures with microspheres larger than 100 microns were used in Interventional Radiology Department before 2012, these consecutive patients were determined as Group A. DEB-TACE procedures in 2012 and thereafter were performed with microspheres sized below 100 microns, and these consecutive patients were determined as Group B.

Blood samples, bleeding profile, alanine aminotransferase (ALT), aspartate transaminase (AST), albumin, total bilirubin and alpha-fetoprotein (AFP) values were investigated before the procedure. Hepatic and mesenteric digital subtraction angiographies with cone-beam computed tomography (not available before 2014) were performed to determine vascular anatomy and tumor-feeding vessels subsequent to the sheath insertion via femoral approach using the Seldinger technique and catheterization of coeliac trunk and superior mesenteric artery. Afterwards, the tumor-feeding arteries were super-selectively catheterized with a microcatheter (1.7 to 2.7 F) to prevent non-target embolization and chemoembolization with doxorubicin-loaded microspheres was performed. The procedure was terminated when the blood flow in tumor feeding arteries was stagnant. Due to the toxic effect, doxorubicin was not given more than 150 mg per one session. Microspheres sized 100–1000 microns (DC-BEAD, BTG plc, Great Britain) and microspheres sized 40 microns, 75 microns and 100 microns (Embozene TANDEM, Boston Scientific, MA) were used for DEB-TACE procedures.

Patients were discharged within 24 hours after procedure. In the first month, patients were followed up ambulatory in terms of postembolization syndrome. For this purpose, based on the Common Terminology Criteria for Adverse Events (CCTAE) [13] classification, patients were questioned about abdominal pain, loss of appetite, weakness, subfebrile fever. Additionally, all patients were investigated for albumin, total bilirubin, ALT, AST values and international normalized ratio of prothrombin time of blood coagulation within 1 month.

Single-session DEB-TACE was performed if the radiological response to treatment was successful, and in the case of residual tumor, the procedure was repeated. Patients were evaluated by dynamic magnetic resonance imaging (MRI) at first month and then periodically at 3-month intervals. Patients, for whom MRI could not be performed, were evaluated by multiphase multidetector computed tomography (CT). MRI 1.5 Tesla device (Signa Excite, General Electric Healthcare, IL) and multidetector CT device (Asteion 4, Toshiba Medical, Japan) were used for follow-up imaging. Dynamic MRI examinations were performed using gadoxetate disodium (Primovist, Bayer Schering Pharma AG, Germany) or gadobutrol (Gadovsit, Bayer Schering Pharma AG, Germany) [14]. In the multiphase CT examinations, ioheksol (Omnipague, General Electric Healthcare, Great Britain), ioversol (Optiray, Mallinckrodt, Quebec) or iobitridol (Xenetix, Guerbet, France) were used. The images before and after treatment were evaluated by a radiologist with 10 years of experience in abdominal radiology according to modified Response Evaluation Criteria In Solid Tumors (mRECIST) criteria [15].

## Statistical analysis

In this study, overall survival (OS), time-to-progression (TTP), progression-free survival (PFS) and adverse events of treatment were evaluated. In addition, overall survival rates and response rates according to mRECIST criteria at 1st, 3rd, and 5th year were calculated. Overall survival and progression durations were calculated by Kaplan-Meier method with log-rank test. The Pearson Chi-Square test for categorical data and Mann-Whitney U test for continuous data were used in the comparative analysis of both groups. Complete response (CR), partial response (PR), stable disease (SD), and progressive disease (PD) were evaluated for each patient according to mRECIST criteria and results were given in percentages. Additionally, an objective response, defined as the sum of CR and PR, was calculated and presented as percentages. Similarly, the ratio of responses to treatment was recorded for the target lesion in terms of mRECIST criteria. For statistical analysis, significance level was accepted as *P <* 0.05. SPSS software version 20.0 (IBM Corp., NY) was used for statistical analysis.

## Results

The demographic and clinical characteristics of the patients who were implemented with this procedure are shown in Table 1. In terms of etiology, in Group A, hepatitis B virus (HBV) was detected in 15 patients (53.6%), hepatitis C virus (HCV) in 7 patients (25.0%) and HBV with HCV (3.6%) in 1 patient. In five patients (17.8%), etiology was not found and these patients were considered to be cryptogenic. In Group B, HBV was detected in 16 patients (53.3%), HCV was found in nine patients (30.0%) and HBV with hepatitis D (3.3%) was detected in one patient (3.3%). In Group B, four patients (13.3%) were considered to be cryptogenic.

**Table 1:** Baseline clinical and demographic characteristics of patients (n = 58).

Parameter	Group A	Group B	*P* value

Gender, n (male/female)	21/7	24/6	0.888
Median age, years (range)	65 (43–76)	66 (46–87)	0.388
Tumor diameter, cm (<3/3-5/>5)	3/15/10	11/11/8	0.205
BCLC(A/B)	12/16	16/14	0.593
Chronic liver disease, n (%)	27 (96.42%)	22 (73.33%)	0.081
Child-Pugh class (A/B)	17/9	17/4	0.391

*Note*: BCLC=Barcelona Clinic Liver Cancer; Mann Whitney U test.

A total of 58 consecutive patients were included in the study. Forty-five (78%) patients were male and no statistically significant difference was found between the two groups in terms of age and gender (*P* = 0.388, *P* = 0.888, respectively) (Table 1). In addition, there were no significant differences between the two groups in terms of Barcelona Clinic Liver Cancer (BCLC) stages, presence of chronic liver disease according to pre-procedural cross-sectional imaging, and Child-Pugh classes (*P* = 0.593, *P* = 0.081, *P* = 0.391, respectively). In Group A, the median lesion size was 51.3 mm (range 25-162 mm) and, in Group B, the median size was 39.6 mm (range 13–80 mm). DEB-TACE procedures for tumors sized below 3 cm were performed due to the inability to demonstrate these lesions on ultrasound scans, tumor location unsuitable for ablation, and patients’ comorbidities. There was no statistically significant difference between the lesion sizes in both groups (*P* = 0.205). In Group A, lesions were located in the right lobe in 19 patients (67.8%), in the left lobe in three patients (10.7%) and both left and right lobes in six patients (21.5%). In Group B, locations of the lesions were right lobe in 22 patients (73.4%), left lobe in four patients (13.3%), and bilobar distribution was detected in four patients (13.3%).

Single DEB-TACE procedure was performed in 20 patients (71.4%) in Group A and in 18 patients (60.0%) in Group B. In Group A, five patients (17.9%) had two sessions, two patients (7.1%) had three sessions, and one patient (3.6%) had six sessions of DEB-TACE. In Group B, nine patients (30.0%) had two sessions, one patient (3.3%) had three sessions, and two patients (6.7%) had four sessions of DEB-TACE procedure. A total of 42 sessions were performed in group A, and in group B, 47 sessions of DEB-TACE were performed. In Group A, median administered cumulative dose of doxorubicin per patient was 84.1 mg (range 25–200 mg), and, in Group B, median dose per patient was 96.7 mg (range 25–250 mg).

**Figure 1:**
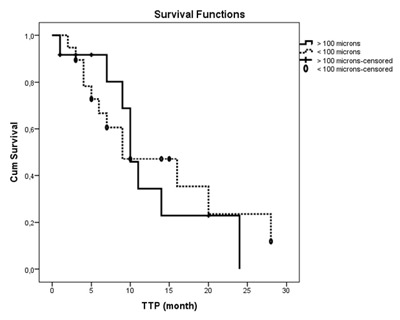
Overall survival analysis of patients in Group A and Group B after DEB-TACE. Note: OS = overall survival (p = 0.190); Kaplan-Meier method with log-rank test.

**Figure 2:**
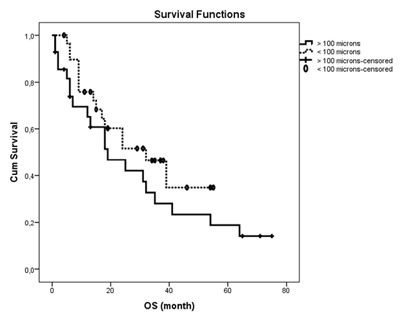
Progression-free survival analysis of patients in Group A and Group B after DEB-TACE. Note: PFS = progression-free survival (p = 0.574); Kaplan-Meier method with log-rank test.

**Figure 3:**
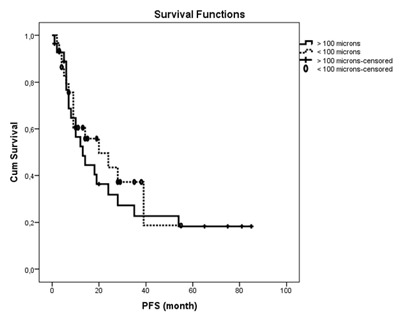
Time-to-progression analysis of patients in Group A and Group B after DEB-TACE. Note: TTP = time-to-progression (p = 0.723); Kaplan-Meier method with log-rank test.

The median follow-up period was calculated as 15 months (range of 1–77 months) for Group A and 24 months (range of 1–75 months) for Group B. There was no statistically significant difference between the two groups in terms of follow-up durations (*P* = 0.227). Table 2 shows the OS, PFS and TTP values for each group. There was no statistically significant difference between two groups in terms of OS and PFS durations (*P* = 0.190, *P* = 0.574, respectively) (Figures 1 and 2). There was no significant difference in TTP durations between the two groups (*P* = 0.723) (Figure 3). In Group A and in Group B, the difference of OS and PFS according to BCLC stage (*P* = 0.178, *P* = 0.205, respectively) or tumor size (*P* = 0.638, *P* = 0.875, respectively) were not statistically significant.

**Table 2:** Time-to-event durations after DEB-TACE.

	Group A	Group B	*P* value

Median OS, months (range)	19 (1–79)	32 (4–60)	0.190
Median PFS, months (range)	13 (1–79)	20 (2–54)	0.574
Median TTP, months (range)	10 (1–24)	9 (2–28)	0.723

*Note*: OS = overall survival, PFS = progression-free survival, TTP = time-to-progression, BCLC = Barcelona Clinic Liver Cancer; Kaplan-Meier method with log-rank test.

Calculated PFS and TTP values were close to each other between Group A and Group B (*P* = 0.299, *P* = 0.715, respectively). There were no statistically significant differences of PFS durations between Group A and Group B in terms of BCLC stages (*P* = 0.183, *P* = 0.449) and tumor size (*P* = 0.265, *P* = 0.829, respectively) (Table 3).

**Table 3:** Target-based time-to-event durations after DEB-TACE.

	Group A	Group B	*P* value

Median OS, months (range)	18 (4–55)	24 (1–75)	0.207
Median PFS, months (range)	17 (3–55)	17 (1–75)	0.299
Median TTP, months (range)	9 (1–24)	10 (3–28)	0.715
BCLC-based median OS, months			
Stage A (range)	12 (5–55)	20 (1–75)	0.183*
Stage B (range)	14 (8–55)	63 (2–65)	
Tumor size-based median OS, months			
<3 cm (range)	32 (5–55)	39 (16–75)	
3–5 cm (range)	9 (8–54)	12 (1–71)	0.449*
>5 cm (range)	18 (4–35)	24 (2–65)	
BCLC-based median PFS, months			
Stage A (range)	12 (5–55)	24 (1–75)	0.265*
Stage B (range)	17 (2–65)	19 (8–60)	
Tumor size-based median PFS (months)			
<3 cm (range)	32 (5–55)	39 (6–75)	
3–5 cm (range)	9 (8–54)	12 (1–71)	0.829*
>5 cm (range)	18 (4–35)	24 (2–65)	

*Note*: OS = overall survival, PFS = progression-free survival, TTP = time-to-progression, BCLC = Barcelona Clinic Liver Cancer; * = significance between all groups; Kaplan-Meier method with log-rank test.

In the evaluation of response to treatment according to mRECIST criteria, there was no significant difference between these two groups (*P* = 0.330) (Tables 4 and 5).

**Table 4:** Response rates after DEB-TACE.

	Group A	Group B	*P* value

First year CR	25.93%	33.33%	0.857*
First year PR	40.74%	30.00%	
First year PD	33.33%	36.67%	
First year OR	66.67%	63.33%	
1–3 years CR	**	16.67%	0.330*
1–3 years PR	7.40%	6.67%	
1–3 years PD	14.81%	20.00%	
1–3 years OR	7.40%	23.33%	
3–5 years PD	3.57%	3.33%	***

*Note*: CR = complete response, PR = partial response, PD = progressive disease, OR = objective response; * = significance between all groups; ** = not observed; *** = not available due to small sample size; Chi-Square test.

**Table 5:** Target tumor-based response rates after DEB-TACE procedure.

	Group A	Group B	*P* value

First year CR	44.82 %	54.84 %	0.563*
First year PR	37.93 %	25.58 %	
First year PD	17.24 %	25.58 %	
First year OR	82.75 %	77.42%	
1–3-year CR	3.44 %	25.81 %	0.280
1–3-year PR	3.44 %	12.90 %	
1–3-year PD	1.79 %	6.45 %	
1–3-year OR	6.90 %	38.71 %	
3–5-year PD	3.57%	3.33%	**

*Note*: CR = complete response, PR = partial response, PD = progressive disease; OR = objective response; * = significance for all groups; ** = not available due to small examples sizes; Chi-Square test.

All DEB-TACE procedures were technically 100% successful. During early follow-ups mortality or major adverse events secondary to treatment were not observed. Grade I or grade II adverse events according to CCTAE classification were observed in all patients (100%). In Group A, in one patient (3.57%), AST value was found to be as high as 443 U/l and ALT as 372 U/L. These changes were considered as Grade III adverse event. Similarly, in one patient (3.33%) from Group B, rising of AST level up to 479 U/L, compatible with Grade III adverse event, was detected. Temporal rising in liver function tests registered in these two patients were reduced to the previous levels in one month without requiring hospitalization. However, there was no difference between the two groups in terms of minor and major complications (*P* = 0.980).

## Discussion

As previously mentioned, HCC is the most common primary malignant tumor of the liver and only 15–20% of patients are suitable for curative treatment at the time of diagnosis. Other patients are diagnosed in the intermediate to terminal stages, and only palliative treatments can be applied to such patients [2,3]. DEB-TACE is a globally accepted palliative treatment method for unresectable HCC [2,4]. However, there is no standardized approach in the literature despite the long-term use of conventional TACE and DEB-TACE therapies with different techniques and approaches. Particularly, the size of the microspheres used for the DEB-TACE procedures vary greatly.

In the first years of DEB-TACE era, 300–500 microns and 500-700 microns sized microspheres were used. Afterwards, it was shown that chemoembolization efficiency was higher in the comparison of 100–300 microns sized microspheres with larger microspheres and the objective response approached up to 50% [16,17,18]. These studies led to the extensive use of 100–300 microns microspheres. The reason why smaller microspheres are more effective is based on the idea that these microspheres can reach to the more distal part of the arteries feeding the tumor, providing a permanent super-selective occlusion of the tumor-feeding arteries. As a result, drug’s direct chemoembolization effect on the tumor increases, and the toxicity of drugs on the non-tumor liver parenchyma decreases by preventing off-target chemoembolization resulting degressive side effects. On the other hand, large microspheres cause earlier stasis as they block the more proximal segments of the tumor-feeding arteries. On the contrary, smaller microspheres do not cause early stasis as they go further distally to the tumor-feeding arteries, and consequently drugs can be delivered directly into the target tumor. Subsequently, this concept was supported by studies in pigs and in explanted livers of six patients who were recipients of liver transplantations [19,20]. It is thought that the theoretical minimal size of the microspheres may be related to the tumor histopathology and the size of the tumor veins in each patient. Studies have shown that the size of intratumoral vascular structures in the HCC is less than 300 microns and usually ranges from 30 microns to 100 microns [19,21]. As a result of different studies in the literature, it is recommended that the lower limit of the microspheres must be between 25 and 50 microns [22,23,24].

Based on this idea, it is expected that DEB-TACE procedures with microspheres which are smaller than 100 microns would be more effective for the HCC treatment. There are a few studies assessing the safety and efficiency of microspheres smaller than 100 microns [8,12,25]. In the present study, the safety and efficiency of the treatment, and patients’ survivals were evaluated comparatively with DEB-TACE procedures performed with doxorubicin-loaded microspheres sized above and below 100 microns.

In terms of safety, no significant difference regarding minor and major complications was observed between the two investigated groups (*P* = 0.98). Although minor complications related to postembolization syndrome were observed in both groups, no major complication was observed. Similar findings were obtained in the safety study on microspheres smaller than 100 microns, reported by Malagari et al. [8], as well as Greco et al. [25], supporting the safety of DEB-TACE with small microspheres. However, Deipolyi et al. in their comparison between 70–150 microns and 100–300 microns sized microspheres, showed that 70–150 microns sized microspheres caused more hepatobiliary side effects although the authors could not explain the exact cause of these complications [12]. In the report of Deipolyi et al. DEB-TACE procedures were performed non-selectively in 9 of 13 patients with hepatobiliary side effects. In these patients, the microspheres were given to the right or left hepatic artery, but not super-selectively to the tumor-feeding artery. The possible cause of high rate of the hepatobiliary side effects may be most likely due to the nonselective administration of small microspheres. In the present study, super-selective catheterization was performed after the tumor-feeding artery was detected and followed by delivering small microspheres into a tumor-feeding vessel. As a result, the side effects of chemoembolization to the intact liver were minimized. Thus, the DEB-TACE performed with microspheres smaller than 100 microns after the super-selective catheterization of the tumor-feeding arteries seems to be a safe treatment for HCC patients.

We didn’t demonstrate statically significant difference in terms of OS, PFS, TTP results between DEB-TACE with smaller or larger microspheres. Larger prospective studies with longer follow-up periods should be performed to better assess long-term results after DEB-TACE with small microspheres for treatment of HCC.

In the evaluation of response to treatment according to mRECIST criteria, there was no significant difference between the two groups. However, Malagari et al. reported that the objective response of the DEB-TACE procedure with microspheres sized 30–60 microns was 68.9% [8], while Greco et al. in the study of DEB-TACE procedure with 40 microns sized microspheres reported the objective response as 72.6% [25]. In these studies, the objective response values were close to the values in the present study and exceeded 50%. As mentioned previously, one of the most important reasons for the use of 100–300 microns microspheres rather than larger microspheres in the DEB-TACE procedures was that the objective response rate approached 50% in extensive studies [16,17,18]. However, in the present study and in other recently published studies, the objective response was approximately 60%–70% in DEB-TACE procedures performed with microspheres smaller than 100 microns. These results relieved that, if supported by more extensive studies, the use of microspheres smaller than 100 microns for DEB-TACE would be widely accepted.

Amesur et al. reported the tumor responses to DEB-TACE procedure with microspheres of 40–120 microns, 100–300 microns and 300–500 microns [11]. In their study, it has been shown that as a result of treatment with 100–300 microns microspheres the tumor shrinkage was more pronounced than after the treatment with other microspheres. No significant difference was observed between 40–120 microns and 100–300 microns microspheres in terms of the decrease in the tumor enhancement. However, the response to the treatment in this study was not based on widely accepted mRECIST criteria, but based on authors’ homemade ones. The absence of the statistical analysis complicates the interpretation of obtained results in a scientific manner.

This study has some limitations. First, it was a single-center retrospective study that reduced its precision. Relatively small number of patients is a second important limitation. However, the number of patients was comparable and the follow-up periods were longer than in the other recently published relevant studies dedicated to DEB-TACE. Larger multicenter studies with longer follow-ups should be performed to better assess DEB-TACE with small microspheres for treatment of HCC. Thirdly, a significant part of the procedures with smaller microspheres were performed under the cone-beam computed tomography guidance; this aspect may affect the selection of the tumor-feeding arteries and as a result may affect the safety and effectiveness of the treatment.

## Conclusion

DEB-TACE procedures performed with doxorubicin-loaded microspheres smaller than 100 microns is an effective and safe method as long as it is applied super selectively and does not cause major complications. However, we failed to demonstrate statically significant difference in terms of OS, PFS, TTP or tumor response (according mRECIST) between DEB-TACE with smaller or larger microspheres. Prospective multicenter studies with a larger number of patients with hepatocellular carcinoma and longer follow-up periods will provide more scientific evidence for the use of doxorubicin-loaded drug-eluting beads smaller than 100 microns.
